# Development and Validation of the Coping Capacity Measurement Scale of Public Health Emergencies in China

**DOI:** 10.3390/ijerph19010094

**Published:** 2021-12-23

**Authors:** Ao Zhang, Hao Yang, Xiang Wu, Xiaowei Luo, Jingqi Gao

**Affiliations:** 1 School of Engineering and Technology, China University of Geosciences, Beijing 100083, China; 2002200096@cugb.edu.cn (A.Z.); wuxiang@cugb.edu.cn (X.W.); 2002190082@cugb.edu.cn (J.G.); 2 Department of Architecture and Civil Engineering, City University of Hong Kong, Hong Kong 999077, China; xiaowluo@cityu.edu.hk

**Keywords:** coping ability, public health emergencies, scale development

## Abstract

Public health emergency coping capacity has been an important direction in crisis research in recent years. The use of the public health emergency coping capacity scale to evaluate the public’s response and feelings regarding public health emergencies is one of the essential ways to improve the effectiveness of public health emergency response. Based on literature research, this paper constructed the theoretical dimension of public health emergency coping ability and completed the development of the items of the initial scale in China. After using SPSS 22.0-conducted exploratory factor analysis, confirmatory factor analysis, and reliability test, the scale dimensions and items were deleted and optimized. The final public health emergency coping capacity measurement scale in China included 12 items and four dimensions. The results showed that the developed scale has high reliability and validity, which is helpful for the relevant personnel to understand the level of public health emergency coping ability and provides an essential basis for timely and accurate emergency prevention and control interventions.

## 1. Introduction

Public health emergencies are unpredictable and can cause significant material and economic losses. They are related to national security, social stability, people’s health, and the long-term and stable development of the world’s economy and society. Coping ability is the ability of individuals to solve problems in everyday life at minimal cost and refers to the adaptability of individuals’ intuition, cognition, emotions, and behaviors [1], and can be reflected by individuals’ behaviors; usually changes in personal ways of thinking, emotions, and behaviors that occur when individuals are challenged by environmental needs, individual needs, expectations, and goals [2]. Current research related to coping capacity focuses on psychology [3,4,5], disaster control [6,7], educational theory and educational management [8]. Although it is difficult to avoid public health emergencies, improving the ability to respond, mastering the relevant knowledge and skills, and taking appropriate measures quickly and in a timely manner will play an invaluable role in the onset of a crisis. Currently, as COVID-19 is a global pandemic public health emergency, whether the public has a high coping capacity during the pandemic determines whether they can quickly adapt to many changes during the outbreak. In the current situation, it seems that the public’s cognition of emergencies is insufficient. From college students with high levels of knowledge and culture, to front-line medical and nursing staff to the general public, all lack emergency response knowledge to varying degrees [9], and their ability to cope with emergencies is generally low. Therefore, it is of great significance to quantitatively evaluate the public’s ability to respond to public health emergencies. However, there are few coping capacity measurement scales for public health emergencies. There are still many questions as to whether the existing coping capacity measurement scales can be fully applicable to the specific environment of public health events.

Public health emergency coping research has attracted much attention since SARS, especially after the COVID-19 outbreak in 2020. Most scholars have discussed the public’s emergency awareness, psychological state and emergency management ability. Rana et al. [10] selected Pakistan as the case study area and designed a 40-item questionnaire, aiming to understand gender differences in COVID-19 risk perception and coping mechanisms; Yin and Ni [11] believed that the effect of COVID-19 on the emotions or behaviors of employees in tourism companies is worth studying, revealing that the intensity of COVID-19 events indirectly affects coping behavior through fear of external threats or psychological security, and that supervisor safety support moderates the effect of psychological safety on this coping behavior. Chen, Zou and Gao [12] investigated the risk and protective factors of psychological distress among Hubei during the peak of the pandemic, and concluded that various stressors related to the new coronavirus outbreak, including risk disclosure, limited access to medical care, inadequate basic supplies, reduced income, overexposure to coronavirus-related information, and perceived discrimination, were associated with psychological stress. Moreover, individual scholars focused on establishing and improving emergency mechanisms and medical protection. Minyoung [13] summarized and analyzed the shortage of healthcare resources, the redistribution of healthcare capacity, reuse of hospitals, and close cooperation between the government and the healthcare commission during the COVID-19 outbreak in South Korea. Another study explored the issue of civic engagement. Zhao et al. [14] conducted in-depth semi-structured interviews with nursing staff to explore the challenges and coping strategies experienced by nursing staff during COVID-19 in China, arguing that different groups encountered different sources of pressure and adopted different coping strategies to fulfill their responsibilities. It is worth mentioning that many studies on COVID-19 have tried to capture the pulse of the nation, and different scholars have used different data sources: Porcher and Renault [15] constructed a novel database containing hundreds of thousands of geotagged messages related to the COVID-19 pandemic sent on Twitter, then analyzed the number of tweets containing various keywords to capture social distancing beliefs and concluded that an increase in the Twitter index of social distancing on the previous day was associated with a decrease in mobility on the following day; Brodeur, Clark, Fleche, and Powdthavee [16] used Google Trends data to test whether COVID-19 and the associated lockdowns implemented in Europe and America led to changes in well-being-related search terms by using difference-in-differences and a regression discontinuity design, and this lead to the conclusion that people’s mental health may have been severely affected by the pandemic and lockdown. It is evident that the outbreak of the COVID-19 pandemic has focused scholars’ attention on the study of response capacity to public health emergencies. However, the development of its measurement tools remains a minority in terms of research.

In terms of coping ability, most of the studies focus on coping ability for a specific group. In contrast, coping ability here primarily refers to addressing all challenges and obstacles in a general and non-directive way. Bode et al. [5] conducted a four-phase group intervention aimed at improving positive coping skills in people aged 50 to 75 years and investigated the positive and negative side effects and differential effects of this educational program. Ito, Seo, Maeno, Ogawa, and Maeno [17] investigated whether Consistency of indicators of stress coping capacity (SOC) could be used to predict depression two years after the beginning of clinical training in Japan through a questionnaire survey. Mahbobeh [18] randomly selected 642 college students to answer two questionnaires to test the validity and reliability of the coping response scale for Iranian college students. The other studies investigate the coping ability of the public, relevant institutions and government departments to natural disasters. Ting, Linsheng, Shaohong, Jiangbo, and Binggan [19] proposed an evaluation method for natural disaster response capability, attempting to quantify the natural disaster response capability to disaster levels and applying it to typhoon prevention and response. Walkling and Haworth [20] conducted in-depth interviews with 12 retired populations in flood risk areas in North Wales, UK, to determine risk perception, coping ability and risk communication preference, to understand the risk experience of the elderly in more detail and provide information for disaster risk reduction (DRR) and communication methods. Chisty and Rahman [6] attempted to understand the vulnerability status of the study area with respect to fire, using a specific vulnerability assessment tool and the existing fire response capabilities of the study area residents. Additionally, a great deal of research in public administration has tried to capture the difference in capabilities and insist on cultural differences: Brian and Shui-Yan [21] fills this gap in the debate by comparing COVID-19 responses among five advanced economies in East Asia: Taiwan, Hong Kong, South Korea, Singapore, and Japan. These studies have specific theoretical and practical significance, and can be used as a reference for public coping capacity for public health emergencies to a certain extent. However, most questionnaires have no measurement indicators, lack modifications and updates after the occurrence of COVID-19, focusing on general coping ability and targeted research.

To sum up, in this study, against the background of the frequent occurrence of global emergencies in recent years and the continued impact of COVID-19 on countries around the world, we attempted to explore the structural model of the public’s ability to respond to public health emergencies from a psychological perspective, using the national situation of China as the basis of the study, develop a scale for investigation, and construct and verify the scale of coping ability of public health emergencies in the background of frequent global emergencies and COVID-19 continues to affect the whole world, to provide a measuring tool for future research on coping ability of public health emergencies. The research results can provide a solid empirical basis for the government and relevant departments to take measures in time.

## 2. Methods

This study discusses the theoretical model of public health emergency coping ability from the perspective of psychology, and was carried out according to the steps of first draft project preparation, scale pretrial and project analysis, exploratory factor analysis, Confirmatory factor analysis and reliability test, which refers to Devellis’ “Scale Development: Theory and Application” [22].

### 2.1. The Theoretical Structure of Public Health Emergency Response Capacity

From the perspective of psychology, this paper refers to the dimension proposed by Epstein and Meier [23], that is, the coping ability factor obtained through factor analysis is composed of one overall factor and six main factors: Global Constructive Thinking; Emotional Coping; Behavioral Coping; Categorical Thinking; Negative Thinking; Superstitious Thinking; Naive Optimism. The details are as follows:

1. Global Constructive Thinking: High scorers are flexible and can adapt their way of thinking to the situation. When the condition is dangerous, they may be pessimistic, but if there is a possible or feasible way to control the problem, they will try to control it. They also accept conditions beyond their control; they accept others as well as themselves; they do not judge others, but they think about how to solve problems.
2. Emotional Coping: High scorers deal with difficult situations in ways that do not create undue stress; they accept themselves and do not take things personally; they are not very sensitive to words such as disapproval, failure and rejection.
3. Behavioral Coping: High scorers are optimistic, enthusiastic, energetic and responsible. Action is usually taken quickly and time is allocated to focus on solving practical problems.
4. Categorical Thinking: High scorers are characterized by extreme thinking, intolerance and distrust of others.
5. Superstitious Thinking: High scorers lack critical thinking and rely too much upon personal judgment.
6. Negative Thinking: High scorers tend to be defensive against threats, and as a result, tend to be pessimistic, unhelpful, and depressed.
7. Naive Optimism: Reasonable optimism is adaptable, energetic and well-liked; on the other hand, the negative side will be simple-minded, the wrong face of adversity.

This paper refers to the six main dimensions of the mature coping capacity measurement scale designed by Epstein and Meier [23], summarizes the literature on the coping capacity of various groups during COVID-19 [24], and combines the characteristics of public health emergencies and anti-pandemic measures taken by China with the coping measures taken by different countries to cope with COVID-19. The ability to cope with public health emergencies was divided into seven dimensions: emotional coping, behavioral coping, absolute thinking, superstitious thinking, negative thinking, pure optimism and disease prevention.

### 2.2. Preparation of Public Coping Capacity for Public Health Emergencies

This paper refers to the Constructive Thinking Inventory (CTI) compiled by Epstein and Meier [23], also known as the “Constructive Thinking Scale”, combined with the characteristics of the paper concept and public health emergencies, for specific deletions and modifications, to obtain a partial coping ability measurement scale. The complete predictive scale included basic population information and three questions, which were general single choice questions, and there are 23 items in the coping ability scale, which is a five-point Likert scale, with a total of 26 items. The specific items and their corresponding dimensions are shown in Table A1 in Appendix A.

### 2.3. Statistical Analysis

In the exploratory factor analysis (EFA), the principal component analysis was adopted in this study to screen factors with eigenvalues greater than 1 in order to provide initial solutions for factor analysis; in addition, the maximum variance rotation method was adopted for factor rotation so that the elements of the columns of the transformed factor loading matrix have the maximum variance after squaring each element while maintaining independence from each other.

In the confirmatory factor analysis (CFA), we performed a common method bias (CMV) analysis, in which all the measures (i.e., the measurement scale items corresponding to all the factors) were placed inside a factor and then analyzed, and if the measures showed that the model fit indicators, such as χ^2^/df, RMSEA, RMR, CFI, etc., could not be met, then the model fit was poor, i.e., it indicated that all the measures were not supposed to belong to the same factor (bad model when placed together), thus indicating that there was no common method bias problem with the data.

## 3. Results

### 3.1. Development of the Initial Scale of Public Health Emergency Coping Capacity

#### 3.1.1. Distribution and Recovery of Pre-Test Questionnaires

The questionnaire was not targeted at a specific group of people and was distributed within mainland China. The questionnaire was prepared and distributed through a questionnaire distribution website, and then disseminated and diffused in the form of websites and social media, and the sample was drawn by snowball sampling. The data was collected from 11 June 2021, 9:18 to 16 June 2021, 12:24. A total of 162 questionnaires were distributed and 162 valid questionnaires were returned, with a valid return rate of 100%. There were 26 items on the scale, and 162 valid questionnaires were much more extensive than three times the number of items, so they were suitable for follow-up analysis.

The scale adopts the five-point scale; that is, there are five options for each item, “strongly inconsistent”, “quite inconsistent”, “consistent”, “quite consistent” and “strongly consistent”, corresponding to 1–5 points, respectively.

#### 3.1.2. Descriptive Statistics

The demographic data of the primary test subjects were analyzed. The results showed that the issues were mainly female, accounting for 56.17% of the whole sample. Most of them were aged between 18 and 25, accounting for 48.77% of the whole sample. The vast majority of the interviewees have a bachelor degree or above, accounting for 78.39% of the full sample. The specific results are shown in Table 1.

#### 3.1.3. Exploratory Factor Analysis

Firstly, KMO and Bartlett sphericity tests were carried out on 23 items, and the results showed that there was a correlation between variables, which was very suitable for factor analysis. Cox et al. showed that the factor load factor should not be less than 0.40, and this paper stipulates that it should not be less than 0.50. The first factor analysis showed that the factor loading coefficients of Q5, Q6, Q17 and Q24 were not up to standard. Therefore, the second factor analysis after deleting these four items showed that the common degree of Q4 was less than 0.4. The third factor analysis was performed after the item was deleted. The following Table 2 shows the results of the KMO and Bartlett sphericity test after the item was deleted.

The results showed that the corresponding P-value of the sphericity test was less than 0.05, and the approximate Chi-square was 956.916 (153 degrees of freedom), which reached a significant level. The KMO value was 0.779, which was more significant than 0.70, indicating a correlation between variables and it was suitable for factor analysis.

**Figure 1:**
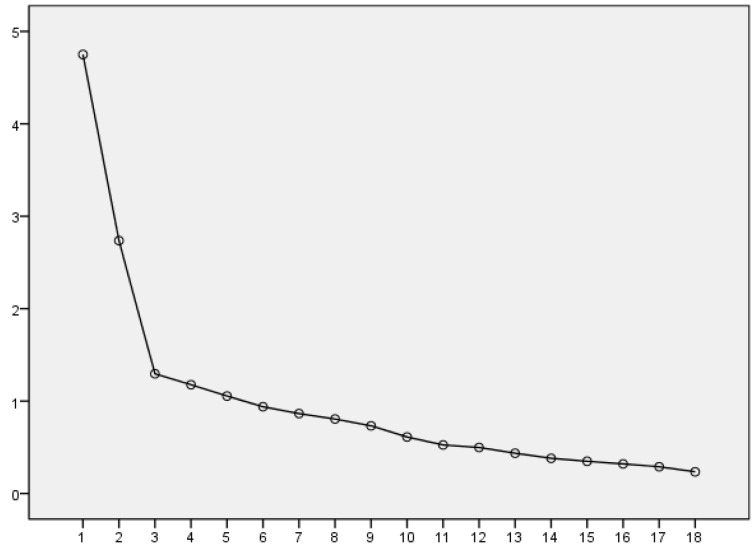
Gravel plot from exploratory factor analysis.

Five common factors were obtained according to variance explained rate and gravel map, and their variance explained rates were 13.944%, 13.442%, 9.903%, 9.039%, 7.757% and 6.785%, accounting for 60.870% of the total, as shown below in Table 3. The eigenvalue gravel plot obtained from the factor analysis is shown in Figure 1.

The commonality of factor analysis results can be used to represent the validity of the scale. The common degree is 0–1, which indicates the ratio of the variance of the original variable determined by the common factor. The closer the commonness value is to 1, showing that the more the original variable information contained in the variable, the better the measurement effect. The common coefficient is generally considered more significant than 0.5, meaning high validity. According to factor analysis results, the items are sorted by dimension and factor load coefficient, and the consequences of specific scale analysis are shown in Table 4.

As can be seen from the table, 18 question items and five factors were finally obtained. The original two dimensions of Categorical Thinking and Disease Prevention are deleted, and the Naive Optimism dimension is renamed as Optimistic Thinking. In contrast, the Negative Thinking dimension is renamed as Pessimistic Thinking. Therefore, the five factors are named Pessimistic Thinking, Behavioral Coping, Emotional Coping, Superstitious Thinking and Optimistic Thinking. In addition, after the selection of items, the remaining items after deletion are reclassified in dimensions. Finally, the initial scale items and dimensions are shown in Table A2 in Appendix A.

#### 3.1.4. Reliance Analysis

To test the validity of the developed scale, this section uses the most common internal consistency reliability to test the reliability of the initial scale of public emergency response capacity after deleting invalid items. The results are shown in Table 5.

From the above table, we can see that the reliability coefficient value is 0.817, and the reliability is more significant than 0.8, which indicates that the study can withstand the repeatability test. The measurement results are accurate, stable and credible. It shows that after the deletion of invalid items, the remaining items should not be deleted, and the next round of evaluation and analysis can be carried out.

### 3.2. Verification of the Initial Scale Public of Coping Capacity to Public Health Emergencies

Based on the analysis results of the initial questionnaire, confirmatory factor analysis was carried out on the initial scale, and reliability and validity analysis was carried out on the coping ability of public health emergencies and its dimensions to ensure the content validity and structural validity of the questionnaire. The scale items were renumbered before analysis. The scale still used the five-point scale method; that is, each item had five options, “strongly inconsistent”, “quite inconsistent”, “consistent”, “quite consistent” and “strongly consistent”, corresponding to 1–5 points, respectively. The higher the score, the higher the capacity to cope with public health emergencies.

The questionnaire was not targeted at a specific group of people and was distributed within mainland China. Similarly, the questionnaire was prepared and distributed through a questionnaire distribution website, and then disseminated and diffused in the form of websites and social media with a snowball sampling of the sample. The data was collected from 24 June 2021, 13:06 until 14 July 2021, 20:29. A total of 556 questionnaires were issued; questionnaires that have been completed in too short or too long a time (according to the number of questionnaire items and topic description judgment, less than 20 seconds or more than 1000 seconds for questionnaire time is too short or too long, respectively), or with the same answers have been deleted. A total of 514 valid questionnaires were recovered, at a recovery rate of 92.4%. There were 18 scale items; as the number of valid questionnaires, 514, was much larger than 10 times the number of items, it was suitable for confirmatory analysis.

The results showed that the samples were mainly female, accounting for 58.02% of the whole sample. Most of them were aged between 26 and 30, accounting for 31.35% of the whole sample. The vast majority of the interviewees have a bachelor degree or above, accounting for 87.57% of the full sample. The specific results are shown in Table 6.

#### 3.2.1. Confirmatory Factor Analysis

Based on the previous exploratory factor analysis, this study uses confirmatory factor analysis (CFA) to verify further the structural validity of the variables in the extensive sample data obtained by formal research. After the first confirmatory factor is completed, the factor load coefficient table (Table 7) and the confirmatory factor fitting results (Table 8) are obtained.

After confirmatory factor analysis, it is found that the load coefficient of several items is not up to standard (less than 0.5), and the validity analysis results also have room for improvement. After combining the content and context of the items, we delete the superstitious thinking dimension and the items D1 and D2 contained in it, and delete the items B1, B4, C3 and E2, and re-conduct the confirmatory factor analysis. The results are shown in Table 9 and Table 10. At this time, the GFI and CFI indexes reached the satisfactory level, and the χ^2^/df, NFI, TLI and RMSEA indexes reached the acceptable level.

After factor covariance analysis, the public’s ability to cope with public health emergencies and its factors all showed significantly (*p* ≤ 0.001), indicating that there was a specific correlation between each factor. Table 11 shows the relationship between factors and factors.

**Figure 2:**
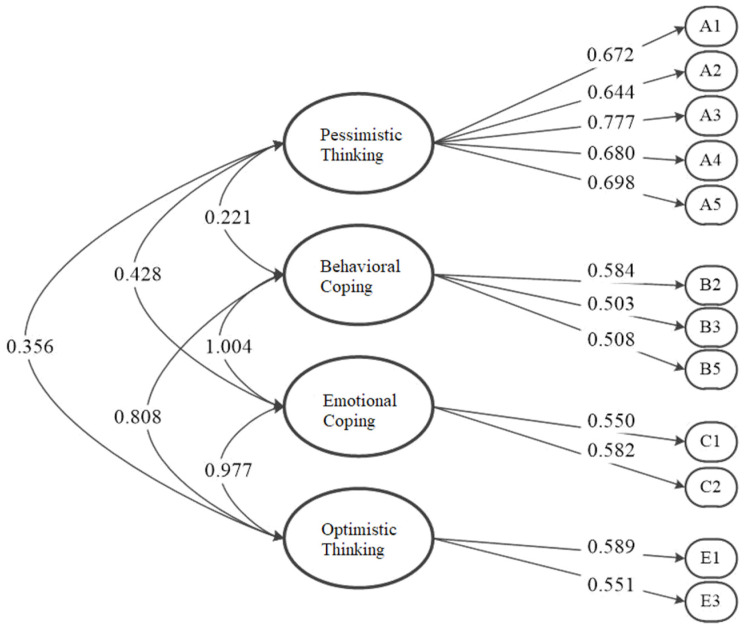
CFA Model Diagram.

According to the estimated load coefficient of factor 10 and covariance of factor 11 in Table, the model is shown in Figure 2.

#### 3.2.2. Reliability and Validity Analysis

The reliability analysis results of SPSS 22.0 software showed that the scale had 12 items and 514 samples, and the Cronbach ‘s α coefficient was 0.786, which was above 0.5, indicating that the scale was reliable.

After Pearson correlation analysis, Table 12 shows the discriminant validity of each dimension of the scale

According to Table 12, the square root value of AVE for each dimension is more significant than the maximum value of the absolute value of the correlation coefficient between the factors, implying that each dimension has good discriminant validity.

In conclusion, the official public health emergency response capacity scale in China includes four dimensions and 12 items. The four dimensions are Pessimistic Thinking, Behavioral Coping, Emotional Coping and Optimistic Thinking. For the formal scale, see Table A3 in Appendix A.

## 4. Discussion

### 4.1. Overview of the Leading Research Results

This study demonstrates the process of compiling and validating the scale of public health emergency response-ability, to obtain a measurement tool that can accurately measure the level of public health emergency response-ability. The final scale consists of four dimensions and 12 items. Its substructure is different from that of the Constructive Thinking Scale (CTI) compiled by Epstein and Meier [23], which may be caused by differences in research contents. Public health emergencies have their unique nature, which seriously threatens human health, causes huge economic losses, and even causes mass panic [25]. Measuring the ability to cope with public health emergencies is not only for the investigation and study of people’s physical or mental health, but also for the adjustment of coping measures to public health emergencies in the future. Therefore, the design of the scale dimensions and scale items integrated the characteristics of public health emergencies. 

The 12-item coping capacity scale of public health emergencies was optimized based on the original 23-item coping capacity scale. The analysis results show that the coping ability scale verified the characteristics of public health emergencies. Adamantios, Marko, Christoph, Petra, and Sebastian [26] suggested that the highest factor loadings could be selected as an assessment metric for a single-item measure, with the 12 items retained all having high factor loadings. In addition, the number of items was significantly reduced compared to the scale of 23 items, making it less time-consuming for respondents to answer the questions and preventing them from becoming bored or tired to a greater extent. It can help emergency managers or other responders quickly access data on people’s ability to respond to public health emergencies so that further targeted measures can be taken.

CTI developed by Epstein and Meier [23] is applicable to measure the coping capacity of the general population in the face of all frustrating challenges, and fewer studies have been conducted for specific domains. Therefore, there is a need to revalidate the resilience scale and test its applicability in the realm of public health emergencies. The 12-item scale is more convenient and has the advantage of being more specific to the field of public health emergencies than other coping capacity scales. The Constructive Thinking Inventory (CTI) developed by Epstein and Meier [23] suggested that traditional measures of coping capacity tend to be one-dimensional, such as depressive sensitivity [27], and do not adequately reflect the richness and complexity of the actual assessment and response process. The 12-item public coping capacity scale developed in this paper is similar to the CTI. The 12-item, six-dimensional scale can well assess public coping capacity during public health emergencies without sacrificing the cognitive and behavioral richness of the process [28]. In conclusion, the 12-item scale developed and validated in this study provides a solid picture of the public’s ability to cope with the various challenges and obstructions that may arise in future public health emergencies.

### 4.2. Theoretical and Practical Significance

This study makes a valuable contribution to current research on health event prevention and control by developing and validating a robust, credible factor structure of the public response capacity to public health emergencies. Unlike previous measurement scales, the scale developed in this study is more appropriate for studying the field of public health emergencies. The scale contributes to the study of response capacity for outbreak prevention and control in the foreseeable future by providing a uniform measure. In addition, the developed and validated scale includes four dimensions: Pessimistic Thinking, Behavioral Coping, Emotional Coping, and Optimistic Thinking, and has high reliability and validity based on 12 question items. The results also indicate that coping competencies are essential for safety management practices during a pandemic, and the validated coping competency scale derived from this study can be used as a primary benchmarking tool between different sectors or firms, thus contributing to the overall safety of the society. The implementation of the coping capacity scale can also provide rich feedback to policymakers and managers to develop public health emergency coping capacity interventions along four dimensions.

### 4.3. Limitations and Future Research Prospects

Although this study has contributed to measuring coping capacity in the field of public health emergencies, future research is needed. This study did not refine the selection of the study population to refer to the public group in general, and future research needs to focus on specific occupational field groups and integrate the characteristics of their work to research coping capacity. In addition, a number of studies have concluded that there are gender differences in stress and coping with stress [29,30], so future studies will be supplemented with corresponding studies that take into account the impact of gender differences on the ability to cope with public health emergencies.

In addition, further validation of the scale should be addressed. The only internal consistency and discriminant values of the subscales were examined. However, neither test–retest reliability nor convergent validity (e.g., correlations with other stress and coping questionnaires) were addressed. Additionally, this study was conducted from June to July 2021, when the new COVID-19 pandemic in China was at a completely different stage. The stress levels and stressors changed dramatically during this phase compared to the beginning of the COVID-19 pandemic in 2020. Therefore, future studies can compare the stress levels and stressors faced by the public in different periods.

During the data analysis, we used factor validity to test the validity of the entire scale. Therefore, in future studies, scholars should continue to explore the above issues in a comprehensive, in-depth, and detailed manner to obtain measurement tools that are more suitable for studying the public’s ability to cope with public health emergencies and to explore further the factor structure of the public’s ability to cope with public health emergencies in a multidimensional and dynamic manner. In addition, an empirical study with a larger sample and demographic difference analysis can be conducted on the scale to validate its applicability further.

## 5. Conclusions

This study first builds a theoretical dimension of the public health emergency coping capability in China based on literature research. After the steps of compiling the first draft project, the preliminary trial of the scale, exploratory factor analysis, verification factor analysis and letter validity test, the scale dimension and question items are deleted and optimized, and the public health emergency coping capacity measurement scale has been developed. The scale includes a total of 12 question items and four dimensions. The four dimensions are Pessimistic Thinking, Behavioral Coping, Emotional Coping, and Optimistic Thinking. The scale has high reliability and validity, which helps relevant personnel to understand the level of public coping to public health emergencies, and to provide a basis for the timely and accurate adoption of targeted emergency prevention and control intervention measures for public health emergencies. However, the research sample size is not large enough and has certain limitations. Future research should expand the sample size and improve the selection of research subjects. Further research can be considered for specific groups, or expand the research scope to further explore the applicability of the scale.

## Figures and Tables

**Table 1:** Demographic data analysis of the initial scale measurements.

Statistical Content	Sample Classification	Number	Percentage (%)	Accumulative Percentage (%)
Gender	Male	71	43.83	43.83
	Female	91	56.17	100.00
Age	Under 18	2	1.23	1.23
	18~25	79	48.77	50.00
	26~30	29	17.90	67.90
	31~40	24	14.81	82.71
	41~50	16	9.88	92.59
	51~60	11	6.79	99.38
	Over 60	1	0.62	100.00
Degree level	Junior high school or below	6	3.70	3.70
	High school	29	17.90	21.60
	Undergraduate	73	45.06	66.66
	Master	51	31.48	98.14
	Dr	3	1.85	100.00

**Table 2:** Results of the KMO and Bartlett spherical tests.

**KMO**		0.779
**Bartlett spherical test**	Approximate chi-square	956.916
	df	153
	Sig.	0.000

**Table 3:** Variance explained rate.

Factor Number	Characteristic Root			Variance Explained Rate			Variance Explained Rate after Rotation		
	CR	VER (%)	VERAR (%)	CR	VER (%)	VERAR (%)	CR	VER (%)	VERAR (%)
1	4.750	26.391	26.391	4.750	26.391	26.391	2.856	15.868	15.868
2	2.736	15.202	41.593	2.736	15.202	41.593	2.763	15.348	31.216
3	1.295	7.197	48.789	1.295	7.197	48.789	1.945	10.804	42.019
4	1.177	6.539	55.328	1.177	6.539	55.328	1.772	9.845	51.864
5	1.055	5.860	61.188	1.055	5.860	61.188	1.678	9.324	61.188
6	0.939	5.215	66.403	-	-	-	-	-	-
7	0.865	4.805	71.208	-	-	-	-	-	-
8	0.806	4.477	75.685	-	-	-	-	-	-
9	0.733	4.069	79.755	-	-	-	-	-	-
10	0.612	3.398	83.153	-	-	-	-	-	-
11	0.525	2.917	86.070	-	-	-	-	-	-
12	0.498	2.766	88.836	-	-	-	-	-	-
13	0.436	2.422	91.258	-	-	-	-	-	-
14	0.381	2.115	93.373	-	-	-	-	-	-
15	0.349	1.937	95.311	-	-	-	-	-	-
16	0.320	1.778	97.089	-	-	-	-	-	-
17	0.289	1.608	98.697	-	-	-	-	-	-
18	0.235	1.303	100.000	-	-	-	-	-	-

**Table 4:** Factor load coefficient after rotation.

Item	Factor Load Coefficient					Common Degree
	Factor 1	Factor 2	Factor 3	Factor 4	Factor 5	
Q10	0.791					0.674
Q9	0.728					0.590
Q12	0.702					0.675
Q7	0.692					0.571
Q15	0.689					0.637
Q26		0.749				0.683
Q18		0.718				0.691
Q19		0.703				0.557
Q22		0.579				0.554
Q23		0.538				0.572
Q8			0.727			0.602
Q20			0.581			0.659
Q25			0.533			0.562
Q11				0.779		0.640
Q21				0.620		0.655
Q13					0.729	0.600
Q16					0.687	0.660
Q14					0.542	0.432

Note: If there are numbers in the table, the absolute value of the factor load coefficient is more significant than 0.5.

**Table 5:** Brief table of Cronbach reliability analysis.

Sample Size	Cronbach’α	Cronbach’α Based on the Standardization Project	Item Number
162	0.817	0.821	18

**Table 6:** Demographic data analysis of the scale measurements.

Statistical Content	Sample Classification	Number	Percentage (%)	Accumulative Percentage (%)
Gender	Male	233	41.98	41.98
	Female	322	58.02	100.00
Age	Under 18	7	1.26	1.26
	18~25	167	30.09	31.35
	26~30	174	31.35	62.70
	31~40	152	27.39	90.09
	41~50	31	5.59	95.68
	51~60	20	3.60	99.28
	Over 60	4	0.72	100.00
Degree level	Junior high school or below	12	2.16	2.16
	High school	57	10.27	12.43
	Undergraduate	388	69.91	82.34
	Master	88	15.86	98.20
	Dr	10	1.80	100.00

**Table 7:** Table of factor loading coefficient.

Factor (Subvariable)	No.	Measurement Item (Dominant Variable)	Coef.	Std. Error	z	*p*	Std. Estimate
Pessimistic Thinking	A1	I tend to classify people as either for me or against me.	1.000	-	-	-	0.666
	A2	I think there are many wrong ways, but only one right way, to almost anything.	1.051	0.086	12.172	0.000	0.640
	A3	When something happens to me, I believe it is likely to be balanced by something bad.	1.305	0.093	14.035	0.000	0.776
	A4	I avoid challenges because it hurts too much when I fail.	1.092	0.085	12.910	0.000	0.689
	A5	When I am faced a new situation, I tend to think the worst possible outcome will happen.	1.158	0.088	13.086	0.000	0.701
Behavioral Coping	B1	I have the habit of deliberately avoiding crowded places.	1.000	-	-	-	0.309
	B2	I will often pay attention to the dynamics of public health emergencies, and if there are signs of an epidemic, I will take precautions.	1.514	0.252	6.008	0.000	0.547
	B3	I only believe in the information released by the official (government, relevant medical institutions).	1.387	0.234	5.936	0.000	0.524
	B4	I have the habit of washing hands with soap or hand sanitizer.	1.413	0.245	5.761	0.000	0.477
	B5	I have the habit of avoiding touching my eyes and nose as much as possible in public.	1.617	0.270	5.990	0.000	0.541
Emotional Coping	C1	When faced with upcoming unpleasant events, I usually carefully think through how I will deal with them.	1.000	-	-	-	0.568
	C2	I would expect the possible consequences of a public health emergency.	0.997	0.100	9.939	0.000	0.540
	C3	I am very concerned about the independent use of personal items like towels, toiletries, etc.	0.828	0.090	9.198	0.000	0.488
Superstition Thinking	D1	I do not believe in any superstition.	1.000	-	-	-	0.473
	D2	I will respond after the disaster news in the media.	1.097	0.121	9.103	0.000	0.642
Optimistic Thinking	E1	If I do well on an important test, I feel like a total success.	1.000	-	-	-	0.569
	E2	I tend to dwell more on pleasant than unpleasant incidents from the past.	0.721	0.101	7.112	0.000	0.417
	E3	I believe that people can accomplish anything they want to if they have enough willpower.	1.023	0.114	8.965	0.000	0.584

**Table 8:** The fitted results for CFA.

Indicators of Fitting		χ^2^/df	GFI	NFI	CFI	TLI	RMSEA
**The fit value**		3.586	0.912	0.818	0.860	0.829	0.071
**Standard value**	**Satisfied**	<5	>0.90	>0.90	>0.90	>0.90	<0.05
	**Acceptable**	3~5	0.85~0.90	0.80~0.90	0.80~0.90	0.80~0.90	0.05~0.08
	**Insufficient**	>5	<0.85	<0.80	<0.80	<0.80	>0.10

**Table 9:** Table of factor loading coefficient.

Factor (Subvariable)	No.	Measurement Item (Dominant Variable)	Coef.	Std. Error	z	*p*	Std. Estimate
Pessimistic Thinking	A1	I tend to classify people as either for me or against me.	1.000	-	-	-	0.672
	A2	I think there are many wrong ways, but only one right way, to almost anything.	1.048	0.085	12.322	0.000	0.644
	A3	When something happens to me, I believe it is likely to be balanced by something bad.	1.295	0.091	14.178	0.000	0.777
	A4	I avoid challenges because it hurts too much when I fail.	1.067	0.083	12.877	0.000	0.680
	A5	When I am faced a new situation, I tend to think the worst possible outcome will happen.	1.142	0.087	13.148	0.000	0.698
Behavioral Coping	B2	I will often pay attention to the dynamics of public health emergencies, and if there are signs of an epidemic, I will take precautions.	1.000	-	-	-	0.584
	B3	I only believe in the information released by the official (government, relevant medical institutions).	0.824	0.102	8.090	0.000	0.503
	B5	I have the habit of avoiding touching my eyes and nose as much as possible in public.	0.938	0.115	8.136	0.000	0.508
Emotional Coping	C1	When faced with upcoming unpleasant events, I usually carefully think through how I will deal with them.	1.000	-	-	-	0.550
	C2	I would expect the possible consequences of a public health emergency.	1.111	0.116	9.606	0.000	0.582
Optimistic Thinking	E1	If I do well on an important test, I feel like a total success.	1.000	-	-	-	0.589
	E3	I believe that people can accomplish anything they want to if they have enough willpower.	0.931	0.112	8.343	0.000	0.551

**Table 10:** The fitted results for CFA.

Indicators of Fitting		χ^2^/df	GFI	NFI	CFI	TLI	RMSEA
**The fit value**		3.978	0.940	0.880	0.907	0.871	0.076
**Standard value**	**Satisfied**	<5	>0.90	>0.90	>0.90	>0.90	<0.05
	**Acceptable**	3~5	0.85~0.90	0.80~0.90	0.80~0.90	0.80~0.90	0.05~0.08
	**Insufficient**	>5	<0.85	<0.80	<0.80	<0.80	>0.10

**Table 11:** The Factor covariance table.

Factor	Factor	Coef.	Std.Error	z	*p*	Std.Estimate
Behavioral Coping	Pessimistic Thinking	0.163	0.030	5.418	0.000	0.428
Behavioral Coping	Emotional Coping	0.150	0.032	4.710	0.000	0.356
Behavioral Coping	Optimistic Thinking	0.093	0.029	3.235	0.001	0.221
Emotional Coping	Pessimistic Thinking	0.278	0.035	7.931	0.000	1.004
Emotional Coping	Optimistic Thinking	0.247	0.034	7.169	0.000	0.808
Optimistic Thinking	Pessimistic Thinking	0.269	0.035	7.768	0.000	0.977

**Table 12:** Discriminant validity: Pearson correlation and AVE square root value.

	Pessimistic Thinking	Behavioral Coping	Emotional Coping	Optimistic Thinking
**Pessimistic Thinking**	***0.698***			
**Behavioral Coping**	0.163	***0.532***		
**Emotional Coping**	0.269	0.520	***0.567***	
**Optimistic Thinking**	0.234	0.428	0.476	***0.570***

Note: The number on the diagonal is the AVE square root value.

## Data Availability

The data presented in this study are available in the Web of Science core database.

## Appendix A

**Table A1:** Predictive test scale.

Content		No.	Question Item
Basic population information		Q1	Gender
		Q2	Age
		Q3	Academic Level (including ongoing studies)
Coping capacity measurement scale	Emotional Coping	Q4	I do not let little things bother me.
		Q5	I tend to take things personally.
	Behavioral Coping	Q6	I am the kind of person who takes action rather than just thinks or complains about the situation.
		Q7	I avoid challenges because it hurts too much when I fail.
		Q8	When faced with upcoming unpleasant events, I usually carefully think through how I will deal with them.
	Categorical Thinking	Q9	I think there are many wrong ways, but only one right way, to almost anything.
		Q10	I tend to classify people as either for me or against me.
	Superstitious Thinking	Q11	I do not believe in any superstition.
		Q12	When something happens to me, I believe it is likely to be balanced by something bad.
	Negative Thinking	Q15	When I am faced a new situation, I tend to think the worst possible outcome will happen.
		Q16	I tend to dwell more on pleasant than unpleasant incidents from the past.
		Q17	I get so distressed when I notice that I am doing poorly in something that makes me do worse.
	Naive Optimism	Q13	If I do well on an important test, I feel like a total success.
		Q14	I believe that people can accomplish anything they want to if they have enough willpower.
	Disease Prevention	Q18	I will often pay attention to the dynamics of public health emergencies, and if there are signs of an epidemic, I will take precautions.
		Q19	I only believe in the information released by the official (government, relevant medical institutions).
		Q20	I would expect the possible consequences of a public health emergency.
		Q21	I will respond after the disaster news in the media.
		Q22	I have the habit of washing hands with soap or hand sanitizer.
		Q23	I have the habit of avoiding touching my eyes and nose as much as possible in public.
		Q24	I am in ill health, and I will choose to seek medical treatment as soon as possible.
		Q25	I am very concerned about the independent use of personal items like towels, toiletries, etc.
		Q26	I have the habit of deliberately avoiding crowded places.

**Table A2:** Initial scale dimensions and question items.

Dimension	No.	Question Item
Pessimistic Thinking	Q10	I tend to classify people as either for me or against me.
	Q9	I think there are many wrong ways, but only one right way, to almost anything.
	Q12	When something happens to me, I believe it is likely to be balanced by something bad.
	Q7	I avoid challenges because it hurts too much when I fail.
	Q15	When I am faced a new situation, I tend to think the worst possible outcome will happen.
Behavioral Coping	Q26	I have the habit of deliberately avoiding crowded places.
	Q18	I will often pay attention to the dynamics of public health emergencies, and if there are signs of an epidemic, I will take precautions.
	Q19	I only believe in the information released by the official (government, relevant medical institutions).
	Q22	I have the habit of washing hands with soap or hand sanitizer.
	Q23	I have the habit of avoiding touching my eyes and nose as much as possible in public.
Emotional Coping	Q8	When faced with upcoming unpleasant events, I usually carefully think through how I will deal with them.
	Q20	I would expect the possible consequences of a public health emergency.
	Q25	I am very concerned about the independent use of personal items like towels, toiletries, etc.
Superstition Thinking	Q11	I do not believe in any superstition.
	Q21	I will respond after the disaster news in the media.
Optimistic Thinking	Q13	If I do well on an important test, I feel like a total success.
	Q16	I tend to dwell more on pleasant than unpleasant incidents from the past.
	Q14	I believe that people can accomplish anything they want to if they have enough willpower.

**Table A3:** Formal scale of public coping capacity to public health emergencies.

Dimension	No.	Question Item
Pessimistic Thinking	A1	I tend to classify people as either for me or against me.
	A2	I think there are many wrong ways, but only one right way, to almost anything.
	A3	When something happens to me, I believe it is likely to be balanced by something bad.
	A4	I avoid challenges because it hurts too much when I fail.
	A5	When I am faced a new situation, I tend to think the worst possible outcome will happen.
Behavioral Coping	B1	I will often pay attention to the dynamics of public health emergencies, and if there are signs of an epidemic, I will take precautions.
	B2	I only believe in the information released by the official (government, relevant medical institutions).
	B3	I have the habit of avoiding touching my eyes and nose as much as possible in public.
Emotional Coping	C1	When faced with upcoming unpleasant events, I usually carefully think through how I will deal with them.
	C2	I would expect the possible consequences of a public health emergency.
Optimistic Thinking	D1	If I do well on an important test, I feel like a total success.
	D2	I believe that people can accomplish anything they want to if they have enough willpower.
